# Improved outcome in acute myeloid leukemia patients enrolled in clinical trials: A national population-based cohort study of Danish intensive chemotherapy patients

**DOI:** 10.18632/oncotarget.12495

**Published:** 2016-10-06

**Authors:** Lene Sofie Granfeldt Østgård, Mette Nørgaard, Henrik Sengeløv, Bruno C. Medeiros, Lars Kjeldsen, Ulrik Malthe Overgaard, Marianne Tang Severinsen, Claus Werenberg Marcher, Morten Krogh Jensen, Jan Maxwell Nørgaard

**Affiliations:** ^1^ Department of Hematology, Aarhus University Hospital, Aarhus, Denmark; ^2^ Department of Clinical Epidemiology, Aarhus University Hospital, Aarhus, Denmark; ^3^ Department of Hematology, The University Hospital Rigshospitalet, Copenhagen, Denmark; ^4^ Stanford University School of Medicine, Stanford, CA, United States; ^5^ Department of Hematology, Copenhagen University Hospital, Herlev, Denmark; ^6^ Department of Hematology, Aalborg University Hospital, Aalborg, Denmark; ^7^ Department of Clinical Medicine, Aalborg University, Aalborg, Denmark; ^8^ Department of Hematology, Odense University Hospital, Odense, Denmark; ^9^ Department of Hematology, Copenhagen University Hospital, Roskilde, Denmark

**Keywords:** acute myeloid leukemia, prognosis, chemotherapy, population-based, trials

## Abstract

Clinical trials are critical to improve AML treatment. It remains, however, unclear if clinical trial participation per se affects prognosis and to what extent the patients selected for trials differ from those of patients receiving intensive therapy off-trial.

We conducted a population-based cohort study of newly diagnosed Danish AML patients treated with intensive chemotherapy between 2000–2013. We estimated accrual rates and compared characteristics, complete remission (CR) rates, and relative risks (RRs) of death at 90-day, 1-year, and 3-years in clinical trial patients to patients treated off-trial.

Of 867 patients, 58.3% (*n* = 504) were included in a clinical trial. Accrual rates were similar across age groups (*p* = 0.55). Patients with poor performance status, comorbidity, therapy-related and secondary AML were less likely to be enrolled in trials. CR rates were 80.2% in trial-patients versus 68.6% in patients treated off- trial. Also, trial-patients had superior survival at 1-year; 72%, vs. 54% (adjusted RR of death 1.28(CI = 1.06–1.54)), and at 3 years; 45% vs. 29% (adjusted RR 1.14(CI = 1.03–1.26)) compared to patients treated off-trial.

Despite high accrual rates, patients enrolled in clinical trials had a favorable prognostic profile and a better survival than patients treated off-trial. In conclusion, all trial results should be extrapolated with caution and population-based studies of “real world patients” have a prominent role in examining the prognosis of AML.

## INTRODUCTION

Randomized controlled trials contribute greatly to the understanding and improvement of the prognosis in acute myeloid leukemia (AML) and are the foundation of establishing new standard treatment regimens and clinical guidelines. However, to be able to extrapolate these data, the characteristics of the trial population must correspond to the general patient's clinical and biological characteristics. [1]

It is well described, that specific patient groups including ethnic minorities, subjects of low socioeconomic status, elderly, and teenagers are underrepresented in oncology clinical trials. [2–4] Although, accrual rates to clinical trials for hematologic malignancies have consistently exceeded accrual rates for solid tumor trials, [1, 5, 6] older patients with acute leukemia remain underrepresented in clinical trials. [1, 5, 7] The proportion of eligible patients not included in any given study is rarely known, but a recent study suggested that clinical AML studies have become more selective over time as a consequence of more stringent in- and exclusion criteria that limit enrollment of patients less likely to respond to therapy. [8]

During the last decade only one study has investigated both accrual rates and differences in characteristics between patients enrolled in AML trials and patients treated off-trial. However, the study included previously untreated, relapsed and primary refractory patients and did not stratify the results by treatment intent or disease state. Moreover, no outcomes were reported. [5]

To investigate how patients selected for clinical trials differ from “real world patients”, we conducted a national population-based cohort study. We determined accrual rates and compared patient and clinical characteristics as well as prognosis in newly diagnosed AML patients enrolled in trials to those treated off-trial at a time where an age-appropriate trial was open. Using the Danish National Leukemia Registry (DNLR)[9], we compared younger and older patients treated with curative intent by intensive chemotherapy within phase III clinical trials in Denmark with patients treated off-protocol according to clinical characteristics, known prognostic factors, complete remission (CR) rate, and survival.

## RESULTS

### Patient characteristics

**Figure 1:**
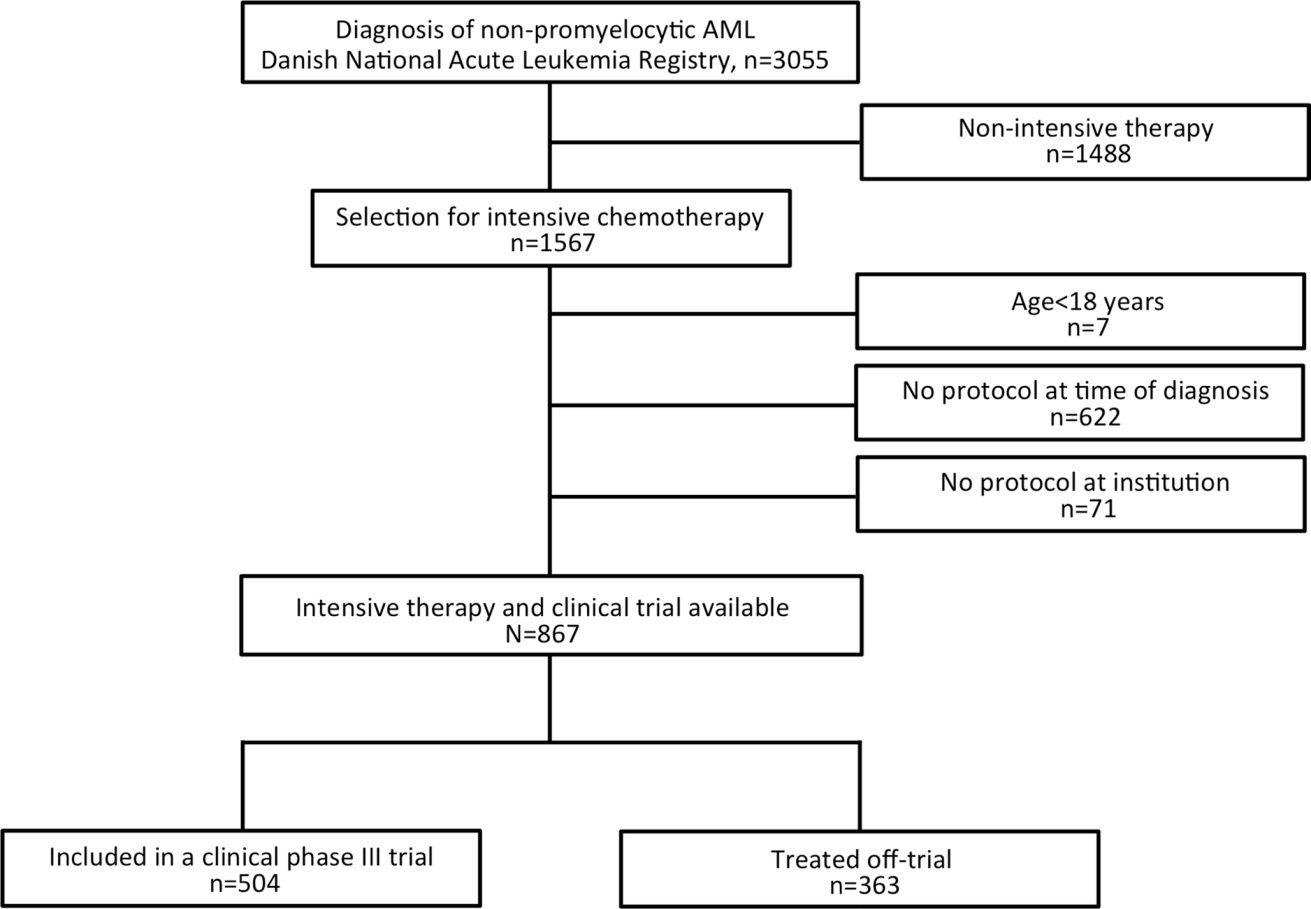
Flowchart of patient selection for the study Patients diagnosed at a time where no age-appropriate trial was open were excluded from the study. All information was obtained from the Danish National Leukemia Registry (DNLR), the Danish National Registry of Patients, and clinical trial office registries. The DNLR contains detailed and valid clinical information on all AML patients (completeness 99.6%) diagnosed in Denmark since 2000. [9]

We excluded patients with non-intensive therapy (best supportive care, *n* = 1141, low-dose chemotherapy, *n* = 347), patients < 18 years (*n* = 7), no protocol at time of diagnosis (*n* = 622), or no protocol at the treating institution (*n* = 71). The selection of the study population is shown in Figure 1.

The final study population consisted of 867 adult AML patients treated with intensive chemotherapy in Denmark between 2002–2013. Of these, 58.3% (*n* = 504) were included in the three UK NCRI-sponsored clinical trials. 190 patients participated in the Medical Research Council (MRC) AML trial 15, (AML15), 161 patients participated in the National Cancer Research institute (NCRI) AML and high risk MDS (myelodysplastic syndrome) trial 16, (AML16), and 153 patients participated in the AML or high-risk myelodysplastic syndrome 17 trial (AML17) [10]. Except for age guidelines and inclusion of high-risk MDS patients in AML16 and AML17, the in- and exclusion criteria for the three NCRI trials were almost identical (Table 1).

**Table 1:** Inclusion and exclusion criteria of the clinical randomized phase III trials in Denmark 2000–2014

	Inclusion criteria	Exclusion criteria
AML15^1^	Diagnosis of AML as defined by the WHO Classification Considered suitable for intensive chemotherapy Age < 60 years, but patients ≥ 60 years eligible if intensive therapy is considered a suitable option Written informed consent	Previous cytotoxic chemotherapy for AML (except for hydroxyurea, or similar low-dose therapy, to control the white count prior to initiation of intensive therapy) Blast transformation of chronic myeloid leukemia (CML) Active malignant disorder Pregnant or lactating
AML16^2^	Diagnosis of AML (excluding promyelocytic leukemia) as defined by the WHO Classification or high risk myelodysplastic syndrome ((RAEB-2, defined as > 10% bone marrow blasts) Age > 60 years, but younger patients eligible if not considered fit for the MRC AML15 trial. Written informed consent	Previous cytotoxic chemotherapy for AML (except for hydroxyurea, or similar low-dose therapy, to control the white count prior to initiation of intensive therapy) Blast transformation of chronic myeloid leukemia (CML) Active malignant disorder (excluding basal cell carcinoma) Patients with abnormal liver function tests exceeding twice the local upper limit of normal are not eligible for the Mylotarg® randomizations Patients with a serum creatinine above the local upper limit of normal are not eligible for the clofarabine randomizations Pregnant or lactating
AML17^3^	Diagnosis of AML as defined by the WHO Classification or high risk myelodysplastic syndrome (RAEB-2; defined as > 10% bone marrow blasts) Age < 60 years, but patients over 60 years eligible if intensive therapy is considered a suitable option Written informed consent	Previous cytotoxic chemotherapy for AML (except for hydroxyurea, or similar low-dose therapy, to control the white count prior to initiation of intensive therapy) Blast transformation of chronic myeloid leukemia (CML) Active malignant disorder Pregnant or lactating Patients with abnormal liver function tests exceeding twice the local upper limit of normal are not eligible for the Mylotarg® randomizations

1 Medical Research Council (MRC) AML trial 15

2 National Cancer Research institute (NCRI) AML and high risk MDS (myelodysplastic syndrome) trial 16

3 AML or high-risk myelodysplastic syndrome 17 trial (AML17)

**Figure 2:**
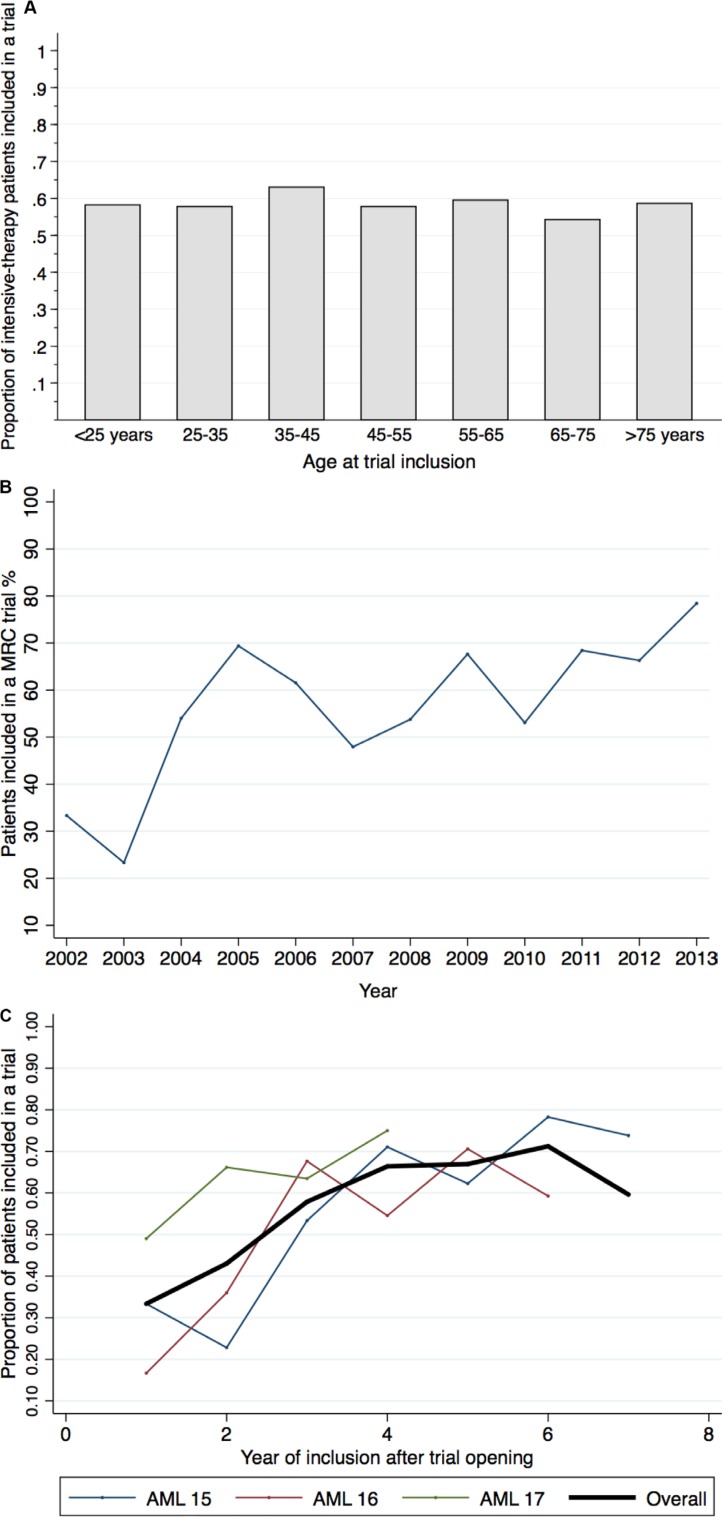
Accrual rates by age and over time Trial inclusion by age groups (**A**), accrual rates per calendar year (**B**), and accrual rates by consecutive years since trial opening (**C**) The accrual rates did not differ between age groups (Cochran-Armitage Test for Trend, *p* = 0.55), but accrual rates increased both with time (B) and the longer a trial remained open (C), (Cochran-Armitage Test for Trend, *p* < 0.001).

The AML15 trial included 61% of patients younger than 60 years eligible for intensive therapy during the study period, compared to 57% in the AML17. The AML16 trial recruited 56% of eligible patients 60 years or older. The accrual rates did not differ between age groups (*p* = 0.55), but for each trial accrual rate increased over time (*p* < 0.001) (accrual rate by year and by patient age, Figure 2). The accrual rate differed significantly between treating institutions, ranging from 41% to 70% (*p* < 0.001).

Overall, median age was 57 years (range 18–80) and 56% were men. The median follow-up time was 532 days (range 0–4388 days). Patients still alive at the end of the study had a minimum follow-up time of 283 days. The characteristics of patients by trial status overall, and by age (< 60 versus ≥ 60 years) are presented in Table 2. Patients enrolled in the trials had fewer adverse prognostic features than patients treated outside the clinical trial setting. In such, patients who presented with worse performance status (PS), white blood cell count (WBC) > 50 mia/l, and lactate dehydrogenase (LDH) > 500 U/l, non-leukemia-related comorbidity, tAML (treatment-related AML), and secondary AML (sAML) were less likely to be enrolled in the clinical trials. However, in multivariate analysis, non-leukemia-related comorbidity was not associated with being treated with standard regimens outside clinical trials. When stratified by age, only tAML had an overall impact on trial inclusion in younger patients. Otherwise, estimates did not differ between age groups (results presented in Table 3).

**Table 2:** Patient characteristics according to age and trial status

	Patients < 60 years, *n* = 507		Patients, ≥ 60 years, *n* = 360	
	Trial	Off-trial	Trial	Off-trial
	*n* = 301 (59.4%)	*n* = 206 (40.6%)	*n* = 203 (56.4%)	*n* = 157 (43.6%)
**Patient characteristics**				
Sex, men, no. (%)	151 (50.2)	109 (51.6)	130 (64.1)	92 (58.6)
Age, median, years, median (range)	48 (18–59)	49 (18–59)	66 (60–79)	66 (60–80)
Numbers of Comorbidities, numbers^1^, (%)				
0	254 (84.4)	162 (78.6)	137 (67.5)	91 (57.9)
1	36 (12.0)	30 (14.6)	47 (23.2)	47 (30.0)
≥ 2	11 (3.6)	14 (6.8)	19 (9.4)	19 (12.1)
WHO PS, no. (%)				
0	147 (48.8)	75 (36.4)	85 (41.9)	41 (26.1)
1	127 (42.2)	89 (43.2)	96 (47.3)	83 (52.9)
≥ 2	27 (9.0)	42 (20.4)	22 (10.8)	33 (21.2)
tAML, no. (%)	8 (2.7)	20 (9.7)	14 (6.9)	12 (7.6)
sAML, no. (%)	18 (6.0)	30 (14.6)	28 (12.7)	35 (23.2)
**Disease characteristics**				
Time to treatment, median (IQR)	2 (1–6)	3 (1–7)	5 (2–9)	4 (1–8)
Blast count marrow, %, median (IQR)	60 (37–81)	62 (40–82)	50 (30–75)	50 (30–75)
Blast count blood, %, median (IQR)	29 (6–67)	35 (10–70)	21 (2–61)	25 (6–58)
White Blood Count, x10^9^/L, median (IQR)	11 (3–37)	17 (3–55)	8 (2–40)	18 (4–67)
Platelet count, x10^9^/L, median (IQR)	55 (33–106)	44 (27–82)	57 (30–93)	52 (29–90)
Lactate Dehydrogenase, U/l^2^, median (IQR)	391 (224–639)	455 (243–1085)	320 (211–520)	491 (266–1000)
Extra-medullary disease, no. (%)	43 (14.6)	32 (15.9)	20 (10.2)	12 (8.1)
Cytogenetics risk group, MRC 2010, no. (%)				
Favorable risk	32 (11.4)	17 (9.4)	8 (4.3)	0 (0.0)
Intermediate risk	199 (71.1)	118 (65.2)	135 (71.8)	110 (77.5)
Adverse risk	49 (17.5)	57 (25.4)	45 (23.9)	32 (22.5)
Missing	21	25	15	15
Cytogenetics, karyotype, no. (%)				
Normal karyotype	140 (50.0)	78 (43.1)	104 (54.7)	72 (50.0)
Abnormal karyotype	140 (50.0)	103 (57.0)	86 (45.3)	72 (50.0)

Abbreviations: WHO PS, World Health Organization Performance Status; tAML, therapy-related AML; sAML, secondary AML; IQR, Interquartile range (25–75 centiles); MRC, Medical Research Council

1 According to the modified Charlson's Comorbidity Index, which includes non-leukemia-related comorbidity

2 Normal range LDH: 105 to 205 U/L for patients < 70 years old, 115 to 255 U/L for patients ≥ 70 years old (randomly missing: *n* = 236)

**Table 3:** Patients and disease characteristics associated with clinical trial enrollment

Patients and Disease Characteristics	Overall		Patients < 60 years		Patients ≥ 60 years	
	Crude OR (95%CI)	Adjusted OR^1^ (95%CI)	Crude OR (95%CI)	Adjusted OR^1^ (95%CI)	Crude OR (95%CI)	Adjusted OR^1^ (95%CI)
Sex, men	1.02 (0.77 − 1.33)	1.01 (0.76 − 1.34)	0.90 (0.68 − 1.31)	0.90 (0.62 − 1.31)	1.26 (0.82 − 1.93)	1.21 (0.77 − 1.90)
Age, years	1.00 (0.99 − 1.01)	1.00 (1.00 − 1.02)	1.00 (0.98 − 1.02)	1.01 (0.99 − 1.03)	0.97 (0.93 − 1.02)	0.97 (0.92 − 1.02)
WHO PS						
0	1	1	1	1	1	1
1	0.65 (0.48 − 0.88)	0.67 (0.49 − 0.91)	0.73 (0.49 − 1.07)	0.78 (0.52 − 0.16)	0.56 (0.35 − 0.90)	0.54 (0.33 − 0.87)
≥ 2	0.32 (0.21 − 0.50)	0.35 (0.23 − 0.56)	0.33 (0.19 − 0.57)	0.35 (0.19 − 0.62)	0.32 (0.17 − 0.62)	0.36 (0.18 − 0.71)
s-AML, Y/N	0.46 (0.30 - 0.69)	0.42 (0.28 − 0.64)	0.37 (0.20 − 0.69)	0.29 (0.16 − 0.57)	0.56 (0.32 − 0.96)	0.53 (0.29 − 0.94)
t-AML, Y/N	0.47 (0.27 − 0.83)	0.49 (0.27 - 0·89)	0.25 (0.11 − 0.59)	0.23 (0.09 − 0.58)	0.89 (0.40 − 1.99)	0.96 (0.40 − 2.30)
White Blood Count, x10^9^/L						
0–2	0.94 (0.62 − 1.42)	0.95 (0.62 − 1.45)	0.83 (0.48 − 1.43)	0.80 (0.45 − 1.41)	1.11 (0.60 − 2.09)	1.10 (0.57 − 2.11)
2–10	1	1	1	1	1	1
10–50	0.83 (0.57 − 1.20)	0.93 (0.63 − 1.36)	0.86 (0.53 − 1.38)	0.95 (0.57 − 1.56)	0.79 (0.44 − 1.42)	0.84 (0.46 − 1.54)
≥ 50	0.56 (0.38 − 0.81)	0.66 (0.44 − 0.97)	0.59 (0.42 − 0.96)	0.66 (0.39 − 1.10)	0.53 (0.30 − 0.95)	0.62 (0.33 − 1.14)
LDH ≥ 500 U/I	0.47 (0.35 − 0.61)	0.48 (0.35 − 0.67)	0.45 (0.31 − 0.65)	0.48 (0.31 − 0.72)	0.45 (0.29 − 0.66)	0.47 (0.28 − 0.80)
Comorbidity, Y/N	0.66 (0.49 − 0.90)	0.79 (0.56 − 1.11)	0.68 (0.43 − 1.07)	0.98 (0.58 − 1.67)	0.66 (0.43 − 1.02)	0.68 (0.43 − 1.08)

Logistic regression analysis, crude and adjusted odds ratios (ORs)

Abbreviations: CI, confidence interval; WHO PS, World Health Organization Performance Status; t-AML, therapy-related AML; s-AML, secondary AML; LDH, lactate dehydrogenase

Age includes as continuous variable, platelet count and marrow blast count included as continuous variables on a logarithmic scale

1 Adjusted for age, sex, WHO PS, sAML, tAML, WBC, LDH, and comorbidity

### Treatment and treatment response

Time to treatment initiation did not differ between patients treated on- or off-trial (median 3 days (interquartile range; IQR 1–7)). Twelve patients (33% of these enrolled on trials) died before treatment initiation. Compared to patients treated off-trial, trial patients were most likely to receive DA/ADE, with or without addition of Gemtuzumab ozogamicin (Mylotarg^®^) or FLT-3 inhibitor (> 92% versus 33%), and FLAG-based regimens (8% versus 4%). Patients treated off-trial more often received cytarabine in combination with idarubicin (56%) or mitoxantrone (7%). Trial patients more often received the planned full-dose second induction regimen than patients treated off-trial (96% versus 88%, *p* < 0.001).

Patients enrolled in trials were also more likely to achieve CR than patients treated outside clinical trials (CR rates and crude ORs, overall and by age are shown in Table 4, Online Only). The overall CR rate in patients enrolled on a trial was 80.2% compared to 68.6% in patients treated off-trial (odds ratio 0.54 (CI = 0.40–0.74)). After adjustment for other factors known to impact CR rates, the effect of clinical trial participation diminished (odds ratio 0.78 (CI = 0.55–1.12)). Though older patients in general were less likely to obtain CR (< 60 years; 80.8% versus ≥ 60 years: 68.3%), stratifying result by age group did not change the conclusions.

**Table 4:** Complete remission (CR) rates and chance of CR (odds ratio; OR) by protocol status, overall and stratified by age

	CR (%)	Crude OR (95%CI)	Adjusted OR^1^(95%CI)
**All patients**			
Trial	404 (80.2)	1	1
Off-trial	249 (68.6)	0.54 (0.40 − 0.74)	0.78 (0.55 − 1.12)
**< 60 years**			
Trial	256 (85.1)	1	1
Off-trial	151 (73.3)	0.48 (0.31 − 0.75)	0.66 (0.40 − 1.12)
**≥ 60 years**			
Trial	148 (72.9)	1	1
Off-trial	98 (62.4)	0.62 (0.39 − 0.96)	0.89 (0.53 − 1.50)

Abbreviations: CI, confidence interval

1 Adjusted for age (continuous variable), sex, white blood cell count (*logarithmically* transformed continuous variable), cytogenetic risk profile (favorable, intermediate, and adverse), WHO PS (0, 1, 2, and ≥ 3), numbers of non-AML-related comorbidities (0, 1 and ≥ 2), sAML, and tAML

Trial patients were more likely to undergo allogeneic hematopoietic stem-cell transplantation (alloHSCT) in first CR (23% versus 17% in patients < 60 years and 23% versus 8% in patients ≥ 60 years). When restricting analysis to patients achieving a CR and adjusting for difference in cytogenetic risk group, comorbidity burden, age, and PS, the likelihood of undergoing transplantation remained higher in trial participants for both younger (adjusted OR 1.93 (CI = 1.07–3.48)) and older patients (adjusted OR 3.88 (CI = 1.58–9.58)).

### Survival, overall and by age

**Figure 3:**
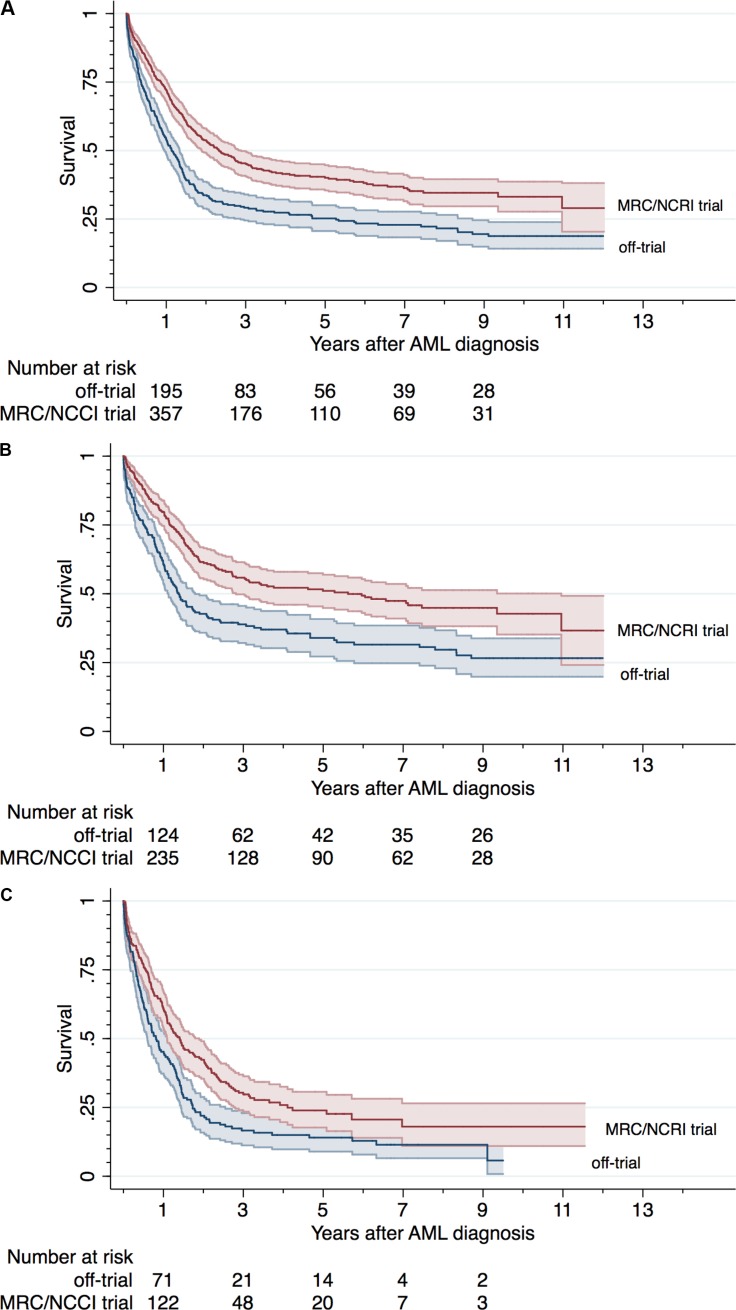
Survival in AML patients by trial status Kaplan Meier Plots with 95%CI bands for the study population overall (**A**), in patients younger than 60 years (**B**), and in patients 60 years or older (**C**).

The crude overall 90-day survival was higher in trial patients than in patients treated off-trial (90% versus 83%, OR 1.72 (CI = 1.22–2.42)), but the difference could be explained by the different distribution of poor risk features (adjusted RR 1.11 (CI = 0.68–1.82)). Crude survival according to trial status and by age are presented in Figure 3 (survival rates) and in Table 5 (crude and adjusted RRs of death at 90 days, 1 year, and 3 years).

**Table 5:** The relative risk (RR) of death within 90-days, 1 year, and 3 years by trial status

	90-days			1-year			3-years		
	Survival (%)	Crude RR (95%CI)	Adjusted RR^1^(95%CI)	Survival (%)	Crude RR (95%CI)	Adjusted RR^1^(95%CI)	Survival (%)	Crude RR (95%CI)	Adjusted RR^1^(95%CI)
**All patients**									
Trial	90	1	1	72	1	1	45	1	1
Off-trial	83	1.72 (1.22 − 2.42)	1.11 (0.68 − 1.82)	54	1.66 (1.38 − 1.98)	1.28 (1.06 − 1.54)	29	1.29 (1.16 − 1.44)	1.14 (1.03 − 1.26)
**< 60 years**									
Trial	94	1	1	80	1	1	56	1	1
Off-trial	85	2.52 (1.44 − 4.38)	1.04 (0.51 − 2.13)	60	1.94 (1.46 − 2.57)	1.44 (1.03 − 2.01)	39	1.39 (1.17 − 1.65)	1.17 (0.99 − 1.39)
**≥ 60 years**									
Trial	84	1	1	61	1	1	30	1	1
Off-trial	80	1.25 (0.81 − 1.95)	0.79 (0.29 − 2.15)	45	1.41 (1.12 − 1.76)	1.21 (0.98 − 1.51)	17	1.19 (1.06 − 1.33)	1.09 (0.98 − 1.22)

Abbreviations: CI, confidence interval

1 Adjusted for age (continuous variable), sex, white blood cell count (logarithmically transformed continuous variable), cytogenetic risk profile (favorable, intermediate, and adverse), WHO PS (0, 1, 2, and ≥ 3), numbers of non-AML-related comorbidities (0, 1 and ≥ 2), sAML, and tAML

One-year survival was superior in clinical trial patients; 72%, compared with 54% in patients treated off-trial (< 60 years: 80% vs. 64%, ≥60 years; 61% vs. 45%), corresponding to an adjusted overall RR of death in patients treated off-trial of 1.28 (CI = 1.06–1.54) compared with clinical trial patients. A difference, albeit smaller, in survival remained present at 3 years (overall adjusted RR 1.14 (CI = 1.03–1.26)).

We performed several sensitivity analyses to test the robustness of our results. In a propensity score-matched survival analysis the estimates did not change our conclusions. Restricting the survival analysis to years with high accrual rate (2004–2012), did not significantly affect results. Also, adjusting for treating institution did not change results in any of the survival analysis. We performed a subgroup analysis restricting the survival analysis to de novo AML patients only (*n* = 605). The results do not deviate from the adjusted results reported for the overall cohort (results not shown).

## DISCUSSION

Results of this study show that patients enrolled in three large UK NCRI AML trials in Denmark had fewer adverse prognostic features compared to AML patients intensively treated off-trial. When adjusting for these differences, however, no differences in complete remission and induction mortality were observed, but both younger and older trial patients had superior survival compared to patients treated off-trial.

To our knowledge, this study, containing virtually complete data on all AML patients treated in Denmark over more than a decade, is the largest study comparing baseline characteristics and prognosis of AML patients treated with intensive chemotherapy on clinical trials to patients treated off-trial. Linkage of high-quality data from national and local clinical and administrative registries offered the opportunity to estimate true accrual rates of the large UK NCRI AML trials in a truly population-based setting. A high quality of data used for the study has previously been confirmed through different validation processes, [9, 11–13] and additional confirmation of trial status using centralized clinical trial office data further minimized misclassification. Essentially complete follow-up data minimized the risk of selection bias in terms of our study aims and adjustment, stratification, and matching for clinically important variables reduced confounding.

Accrual rates of the UK NCRI AML trials activated in Danish institutions match recent accrual rates reported for HOVON AML trials (68% in patients 18–40 years, and 57% in patients 41–60 years) [14] and exceed accrual rates of 44–45% previously reported by two AML studies, [1, 5] In contrast to the previous studies, we did not find accrual rates in intensively treated patients to vary by sex or to decrease with age. [3, 7, 14–16] We speculated that the high accrual rate in our cohort reflect less restrictive in- and exclusion criteria in UK NCRI AML trials. Also, most prior studies comparing characteristics and prognosis in cancer patients treated on and off-trial included patients regardless of treatment intent. Historically, most trials have investigated intensive therapy, thus comparing trial patients to all patients treated outside off clinical trials, would clearly generate bias towards a greater difference in baseline characteristics and outcome between on- and off-trial patients and may partly explain lower accrual rates in some previous reports. Herein, we restricted the study population to patients homogeneously treated with intensive therapy and harmonized according to treatment intent and period of diagnosis.

In addition, clinical trial enrollment in our cohort was not negatively affected by non-leukemia-related comorbidities, again reflecting nonrestrictive inclusion criteria for UK NCRI AML trials. This enables and encourages the physicians to enroll intensive therapy eligible patients with comorbidity into these trials and permits readily translation of clinical trial results to broader AML cohorts. Similar to previously observed, presence of sAML or tAML negatively impacted enrollment into clinical trials, [12] at least in part, due to risk of excessive anthracycline exposure. These findings limit our understanding on therapeutic effects in these subgroups of patient, when only focusing on clinical trial results.

Information regarding the generalizability of clinical trial results is important and can only be obtained from observational studies that include and compare patients treated on trial as well as off-trial. Our study shows that selection bias is present in the MRC trials enrollment within Danish centers, though most other trials have even stricter in- and exclusion. Therefore, It is reasonable to speculate that selection bias is introduced into almost every trial. Repeating this study in a setting with less inclusive trials would likely lead to lower accrual rates and an even more pronounced difference in characteristics and outcome between trial patients and off-trial patients. This hamper the external validity of these trials makes it crucial to consider patient characteristics of the trial population before referring results and treatment regimens to “real world patients.”

Generally, AML requires rapid initiation of treatment to avoid early deaths. Clinical trial screening can be an intense and time consuming process, which may impact time to treatment initiation or affect accrual rate in patients most in need for rapid therapy. Importantly, time to treatment did not differ between patients treated on and off- trial. It remains unclear, though, why patients with worse PS and elevated baseline WBC were less likely to participate in clinical trials, although physician preference may have influenced this decision. Another possible explanation is that patients acutely affected by disease or complications would be less likely to read the required material, understand trial concepts, and provide informed consent.

It has previously been suggested that participation in a trial may confer a survival benefit independent of patient and disease characteristics as well as treatment. [17–20] Differences in outcome between patients treated in clinical trials compared to patients treated off trial can be due to three main factors; difference in baseline characteristics, difference in treatment modalities, and/or the effect of being enrolled in a trial (placebo effect, superior observation, and supportive care). Though, we found a significant difference in baseline characteristics in patients enrolled in trial and patients treated off-trial, adjusting or matching showed that these factors did not fully explain the superior survival found in trial patients. The UK NCRI AML trials did not dictate additional outpatient visits, specific supportive care, or treatment of complications and side effects different from standard treatment off-protocol that could explain the superior survival.

The rather complex multifactorial designs of the UK NCRI trials allow numerous randomized questions to be addressed in one single trial, but so far, only a few experimental treatment arms have demonstrated improved overall survival compared to the control treatment (Supplementary Table S1). In our study, chemotherapy regimens differed between the two groups as more patients treated off-trial received idarubicin-containing induction therapy, whereas trial-patients most often received daunorubicin plus cyterabine with or without etoposide (DA or ADE). The AML15 showed that DA and ADE are comparable. [21] Also, mitoxantrone-containing induction regimens have been found to be comparable to DA in respect of CR-rates and OS. [22] Gemtuzumab ozogamicin more often used in trial-settings showed a marginal overall survival benefit at 3-years in older patients in the AML16, [23] but in AML15, the survival benefit was only found in favorable risk AML. [24] When comparing FLAG-Ida to DA/ADE, only the few patients receiving a total of 4 courses had a significantly better overall survival [21].

More patients treated on-trial had a alloHSCT in first CR compared to off-trial patients. AlloHSCT was not a part of the randomization in the UK NCRI trials, but Russell et al. recently showed superior overall survival in reduced intensity conditioning alloHSCT recipients enrolled in AML15 for the subgroup of patients receiving a matched sibling graft compared to patients receiving consolidation chemotherapy [25].

Whether the survival benefit found in this study between patients treated off-trial and trial-patients regardless of treatment arm was due to trial inclusion in itself, due to difference in treatment especially higher frequencies of GO and alloHSCT, or due to unmeasured confounding cannot be adequately answered by this study.

Reasons for declining participation in clinical trials are multifactorial and some patients may reject trial inclusion due to uncertainty of getting an adequate therapy. To improve general treatment outcomes in AML patients, including patients into investigational trials is crucial. Our observations that AML patients enrolled in randomized clinical trials had, at least, comparable outcomes to patients treated off-trial, could help assist patients in the decision process of considering enrollment on a clinical trial.

Our study has limitations. Though we excluded all patients diagnosed when no suitable trial was open, we did not take into account shorter periods of trial closure, due to local reasons and issues, and to trial amendments. Since this type of bias will decrease accrual rates and lead relative estimates toward no association, this selection is likely of little significance. Importantly, the DNLR does not capture the reason for treating a patient off-trial. In relation to the UK NCRI AML trials in Denmark, reasons for opting-out may be disease-related, trial-related (exclusion criteria, fear off additional side effect from investigational drugs), due to physician and institutional factors, or patient-related factors (reluctance against trials or socioeconomic factors). Still, socioeconomic factors are thought to play a lesser role compared to previous findings in American studies, [3, 26] since health care including trial enrollment is free to all Danish citizens.

In summary, AML patients enrolled in the UK NCRI AML trials in Denmark had a more favorable profile than patients treated with standard intensive therapy regimens off-trial. Even after adjustment for the prognostic factors unevenly distributed between the two groups, trial participation remained associated with a significantly better survival; showing an 28% decreased risk of death at 1-year and an 14% decreased risk of death at 3-years. Since patient characteristics and outcomes in trial patients were not generalizable to intensive therapy patients, population-based studies continue to have an important role in examining aspects of treatment and prognosis of AML patients.

## MATERIALS AND METHODS

We analyzed a cohort of all adult non-promyelocytic AML patients diagnosed in Denmark between January 2000 and December 2013. The Danish population (approximately 5.7 million people) [27] is entitled free access to tax-supported medical health care. During the study period, AML patients were treated with intensive chemotherapy at only five highly specialized institutions. No treatment of hematological cancers takes place in private hospitals, which ensure uniform high-standard supportive care and treatment regimens to all patients. We excluded non-intensive chemotherapy patients with intensive therapy being defined as allocation to a remission induction regimen and an anthracycline or anthracycline-related compound (intention-to-treat objective). Also, we excluded patients if no age-appropriate trial was open at time of diagnosis (Flow chart for study selection, Figure 1).

### Clinical trials

During the study period, newly diagnosed AML patients eligible for intensive therapy were enrolled into three randomized phase III trials (Table 1). Patients not fulfilling the age criteria could be included if the treating physician found the treatment offered by the protocol suitable for the given patient. The MRC AML15 was designed for patients younger than 60 years and opened at four out of five specialized centers in Denmark from 2002. The last patient was included in 2009. AML16 included primarily patients 60 years or older and was open at all specialized centers in 2007–2012. The AML17 replaced AML15 and opened in 2010 at all specialized centers and was still recruiting at end of the study period. The investigational treatment and a summary of the published results of the three trials are shown in Supplementary Table S1.

### Clinical data

We obtained all clinical information from the Danish National Leukemia Registry unless otherwise noted. The registry contains detailed and valid clinical information on all AML patients (completeness 99.6%) diagnosed in Denmark since 2000. [9]

Date of diagnosis was defined as date of first diagnostic bone marrow examination. Cytogenetic results were grouped according to MRC's 2010 revised criteria. [28] We validated information on clinical trial participation using centralized trial clinical office data. We obtained information on non-leukemia-related comorbidity (according to a modified version of the Charlson comorbidity Index; no comorbid disease, one comorbid disease, or ≥ 1 comorbid disease) from the Danish National Registry of Patients. [11]

### Treatment outcome

CR criteria were based on international consensus criteria for morphological CR after two cycles of induction chemotherapy. [29] We obtained information on all-cause mortality and immigration from the Civil Registration System. The Civil Registration System uses a unique civil registration number to track daily updated information on vital status and residence on all Danish residents. [30]

### Statistical analyses

We stratified descriptive data by clinical trial status and age. We computed prevalence of clinical trial accrual for all AML patients, and trends in participation by age, according to year of trial inclusion, and by treating institution (Cochran-Armitage Test for Trend). Treatment modalities and accessible trials differed for younger or older patients. For comparable groups, we therefore stratified all analyses by age ≤60 and >60 years since this cut-off was used in the UK NCRI AML trials. Trial participation was used as a reference, and all estimates included corresponding 95% confidence intervals (CIs).

To assess the association between characteristics and clinical trial inclusion as well as the likelihood of allogeneic hematopoietic stem-cell transplantation performed in first CR by trial status, we used logistic regression analysis (Crude and adjusted odds ratios (ORs)).

Patients were followed from date of diagnosis until death, immigration, or end of follow up (Sep 22^nd^, 2014). Overall survival was described using Kaplan-Meier curves with 95% CI bands. To fit generalized linear models for crude survival we used a pseudo value approach and calculated pseudo values and crude and adjusted relative risks (RRs) of death at 90 days, 1 year, and 3 years. [31–33] We adjusted for age, sex, cytogenetics, WHO PS, comorbidity, WBC, sAML, and tAML.

To test robustness of the results, we repeated survival analyses adjusting and matching for propensity scores predicting probability to enter a trial conditional on age, sex, cytogenetics, PS, comorbidity, WBC, sAML, and tAML (552 patients matched 1:1 without replacement). [34, 35] Accrual rates varied between treating institutions, we therefore repeated all analyses with adjustment for treating institution. Also, we restricted survival analyses to years with high accrual rate. We additionally performed a subgroup analysis restricting the survival analysis to de novo AML patients only.

The study was approved by the Danish Data Protection Agency (j.nr. 2012–41–0878) and the National Board of Health (j.nr.3–3013–158). Analyses were conducted using Stata version13.1 software (STATA Corp, College Station, TX, USA).

## SUPPLEMENTARY MATERIALS TABLES


