# Supplementary material for: Improved outcome in acute myeloid leukemia patients enrolled in clinical trials: A national population-based cohort study of Danish intensive chemotherapy patients
Source: Oncotarget. 2016 Oct 6;7(44):72044–56. doi: 10.18632/oncotarget.12495 (PMC5342143; doi:10.18632/oncotarget.12495)
Supplement: Supplementary file 1 [file oncotarget-07-72044-s001.pdf]

Improved outcome in acute myeloid leukemia patients enrolled in clinical trials: A national population-based cohort study of Danish intensive chemotherapy patients

Supplementary Materials

Supplementary Table S1: Main treatment arms and results of the MCR trials in Denmark

	Treatment randomizations	Main results
AML15 [1–3]<br>For patients < 60 years*	Remission-induction therapy:<br>2 courses of DA versus ADE or FLAG-Ida<br><br>Consolidation therapy:<br>Mitoxantrone/cytarabine (MACE-MidAC) or high-dose cytarabine 3 g/m^2 or 1.5 g/m^2<br><br>Adjuvant therapy:<br>+/– gentuzumab ozagamicin (GO; mylotarg®)<br>FLT3-ITD positive patients CEP-701	DA versus ADE:<br>No difference in CR, RFS, or OS<br>ADE versus FLAG-Ida: reduces relapse risk, but no survival benefit<br>FLAG-Ida:<br>Better OS for patients receiving a total of 4 courses, when given FLAG-Ida as induction as compared to ADE/DA<br>MACE/MidAc versus. high-dose cytarabine (1.5/3.0 g/m^2) :<br>Similar overall outcomes, although MACE/MidAc favourable in high risk patients.<br><br>GO:<br>No overall survival benefit of GO, but survival benefit in subgroup analysis of favorable risk patients
AML16 [4–7]<br>For patients > 60 years**	Remission-induction therapy:<br>DA or DClo, with or without GO 3 mg/m^2 on day 1 of course1<br>DA vs ADE with or without ATRA from d1-60<br><br>Consolidation therapy:<br>In CR after course 1:<br>DA 2+5 versus no consolidation +/– Azacytidine maintenance (1 year)<br>In CR after course 2: DA 2+5 +/– Azacytidine maintenance (1 year)<br><br>AlloHCT (clinician's choice):<br>Induction therapy +/– DA 2 + 5 followed by alloHCT	GO:<br>Better OS in GO vs no GO<br><br>Etoposide or ATRA:<br>No benefit of Etoposid or ATRA to DA<br>DA +/– Clofarabine:<br>DA and DA+Clofarabine give similar outcomes with equivalent toxicity<br><br>Azacytidine:<br>Maintenance therapy did not improve survival<br><br>2 versus 3 courses:<br>An additional course 3 did not improve survival
AML17 [8–10]<br>For patients < 60 years*	All patients:<br>DA 90 mg/m^2 versus 60 mg/m^2 (day 1,3, 5 of first induction course), followed by 50mg/m2 (day 1,3, 5) in course 2.<br>Ara-C was given 100 mg/m^2 12 hourly days 1–10 (course 1) and days 1–8 (course 2)<br>+/– GO 3 mg/m^2 versus 6 mg/m^2 on day 1<br><br>NPM1+ AML:<br>DA versus ADE and ATRA versus no ATRA<br>DA +/– mTor or FLT3 Inhibitor CEP-701<br><br>No-CBF, NPM1+, poor risk AML:<br>DA +/– mTor<br><br>No-poor risk AML:<br>3 versus 4 cycles of chemotherapy:<br>1 versus 2 Ara-C 3 g/m^2<br><br>Poor risk AML:<br>Clofarabine versus Flag-Ida as consolidation	DA 90 mg/m^2 versus DA 60 mg/m^2:<br>No difference in CR (90 mg/m^2 81% versus 60 mg/m^2 84%; OR 1.21(0.90–1.64), $p = 0.1$). No difference in survival overall or in any of the subgroups<br><br>GO:<br>no benefit in giving a single 6mg/m2 dose of GO compared with 3 mg/m^2<br><br>NPM1+ AML:<br>ADE versus DA:<br>No differences in early mortality or overall survival at 2 years<br><br>ATRA versus no ATRA:<br>No difference in OS<br><br>Lestaurinib:<br>Addition of Lestaurtinib did not improve outcome

Abbreviations: GO, gemtuzumab-ozogamicin, Mylotarg®, RFS, relapse-free survival; OS, overall survival; CR, complete remission
*Older patients eligible if intensive therapy is considered a suitable option
**Younger patients eligible if intensive therapy is considered a suitable option

REFERENCES

1. Burnett AK, Russell NH, Hills RK, Hunter AE, Kjeldsen L, Yin J, Gibson BE, Wheatley K, Milligan D. Optimization of chemotherapy for younger patients with acute myeloid leukemia: results of the medical research council AML15 trial. Journal of clinical oncology. 2013; 31:3360–3368.
2. Burnett AK, Hills RK, Milligan D, Kjeldsen L, Kell J, Russell NH, Yin JA, Hunter A, Goldstone AH, Wheatley K. Identification of patients with acute myeloblastic leukemia who benefit from the addition of gemtuzumab ozogamicin: results of the MRC AML15 trial. Journal of clinical oncology. 2011; 29:369–377.
3. Russell NH, Kjeldsen L, Craddock C, Pagliuca A, Yin JA, Clark RE, Howman A, Hills RK, Burnett AK. A comparative assessment of the curative potential of reduced intensity allografts in acute myeloid leukaemia. Leukemia 2015; 29:1478–1484.
4. Burnett AK, Russell NH, Hills RK, Kell J, Freeman S, Kjeldsen L, Hunter AE, Yin J, Craddock CF, Dufva IH, Wheatley K, Milligan D. Addition of gemtuzumab ozogamicin to induction chemotherapy improves survival in older patients with acute myeloid leukemia. Journal of clinical oncology : official journal of the American Society of Clinical Oncology. 2012; 30:3924–3931.
5. Hills RK, Friis LS, Kjeldsen L, Milligan D, Hunter AE, Lazenby M, Gilkes AF, Bowen D, Russell N. .The ATRA Question In AML: Lack Of Benefit Overall Or In Any Molecular Subgroup In The NCRI AML16 Trial. Blood. 2013; 122:abstract 493.
6. Burnett A, Russell N, Freeman S, Kjeldsen L, Milligan L, Pocock C, Cahalin P, Kell J, Dennis M, Hills R. A comparison of limited consolidation chemotherapy or not, and demethylation maintainance or not in older patients with AML or high risk MDS: long term results of the P UK NCRI AML16 trial. Heamatologica. 2015; 100 (s1) abstract 513.
7. Russell N, Burnett A, Kjeldsen L, Milligan D, Cahalin P, Kell J, Dennis M, Hills R. A Comparison of daunorubicin/Ara-C versus daunorubicin/clofarabine as induction treatment in older patients with AML and high risk MDS: long term results of the UK NCRI AML16 trial in 806 patients . Haematologica. 2015; 100 (s1) abstract 514.
8. Burnett AK, Russell N, Hills RK, Cavenagh J, Kell J, Jones G, Khwaja A, McMullin MF, Dennis M, Milligan D, Clark RE. A Comparison of Single Dose Gemtuzumab Ozogamicin 3mg/m2 and 6mg/m2 Combined with Induction Chemotherapy in Younger Patients with AML: Data from the UK NCRI AML17 Trial. Blood. 2014;124: abstract 2308.
9. Burnett AK, Russell NH, Hills RK, Kell J, Cavenagh J, Kjeldsen L, McMullin M, Cahalin P, Dennis M, Friis L, Thomas IF, Milligan D, Clark RE. A randomized comparison of daunorubicin 90 mg/m2 vs 60 mg/m2 in AML induction: results from the UK NCRI AML17 trial in 1206 patients. Blood 2015; 125: 3878–3385.
10. Knapper S, Hills RK, Cavenagh JD, Kjeldsen L, Hunter AE, Clark RE, Dennis M, Milligan D, Levis MJ, Russell N, Burnett AK. A Randomised Comparison of the Sequential Addition of the FLT3 Inhibitor Lestaurtinib (CEP701) to Standard First Line Chemotherapy for FLT3-Mutated Acute Myeloid Leukemia: The UK Experience. Blood. 2014;124: abstract 3736.
